# Transposon Mutagenesis of *Pseudomonas syringae* Pathovars *syringae* and *morsprunorum* to Identify Genes Involved in Bacterial Canker Disease of Cherry

**DOI:** 10.3390/microorganisms9061328

**Published:** 2021-06-18

**Authors:** Helen C. Neale, Michelle T. Hulin, Richard J. Harrison, Robert W. Jackson, Dawn L. Arnold

**Affiliations:** 1 Centre for Research in Bioscience, Faculty of Health and Applied Sciences, Frenchay Campus, The University of the West of England, Bristol BS16 1QY, UK; Helen2.Neale@uwe.ac.uk; 2 NIAB EMR, East Malling ME19 6BJ, UK; Michelle.Hulin@tsl.ac.uk (M.T.H.); Richard.Harrison@niab.com (R.J.H.); 3 NIAB, Cambridge CB3 0LE, UK; 4 Birmingham Institute of Forest Research (BIFoR), University of Birmingham, Birmingham B15 2TT, UK; R.W.Jackson@bham.ac.uk; 5 School of Biosciences, University of Birmingham, Birmingham B15 2TT, UK; 6 Harper Adams University, Newport TF10 8NB, UK

**Keywords:** transposon mutagenesis, bacterial canker, *Pseudomonas syringae*, pathogenicity

## Abstract

Bacterial canker of *Prunus*, affecting economically important stone fruit crops including cherry, peach, apricot and plum, is caused by the plant pathogen *Pseudomonas syringae* (*P.s*.). Strains from two pathovars—*P.s.* pv. *syringae* (*Pss*) and *P.s*. pv. *morsprunorum* race 1 (*Psm*R1) and 2 (*Psm*R2)—in three phylogenetically distant clades have convergently evolved to infect *Prunus*. The bacteria enter woody tissues through wounds and leaf scars, causing black necrotic cankers. Symptoms are also produced on blossom, fruit and leaves. Little is known about the mechanisms *P.s.* uses to colonise tree hosts such as *Prunus*. Here, we created transposon (Tn) mutant libraries in one strain of *P.s.* from each of the three clades and screened the mutants on immature cherry fruit to look for changes in virulence. Mutants (242) with either reduced or enhanced virulence were detected and further characterised by in vitro screens for biofilm formation, swarming ability, and pathogenicity on leaves and cut shoots. In total, 18 genes affecting virulence were selected, and these were involved in diverse functions including motility, type III secretion, membrane transport, amino acid synthesis, DNA repair and primary metabolism. Interestingly, mutation of the effector gene, *hopAU1*, led to an increase in virulence of *Psm* R2.

## 1. Introduction

*Pseudomonas syringae* (*P.s.*) is a bacterial plant pathogen made up of over 60 pathovars (pv) which cause disease in over 180 plant species including many important crops. Bacterial canker caused by *P.s.* is the leading cause of disease in cherry and there are as yet no effective control measures [1]. Bacterial canker of *Prunus* which attacks the economically important crop species cherry, peach, apricot and plum is caused by members of at least three clades of *P.s.*, *P.s.* pv. *syringae* (*Pss*) and *P.s.* pv. *morsprunorum* race 1 (*Psm* R1) and 2 (*Psm* R2). The *Rsm* races are found in two different phylogroups (R1 in P3, R2 in P1) [1]. Bacterial canker has been reported to result in tree losses of up to 75% [2] and is an annual problem. The pathogens occupy two niches including an epiphytic phase where the bacteria remain on the surface of the plant, and an endophytic phase in the intercellular spaces within plant tissues [3]. To reach the intercellular space in woody tissue, the bacteria enter through wounds and leaf scars in the winter and lie dormant until the spring when black necrotic cankers develop [4]. Blossom, fruit and leaves are all affected by the disease and display a range of necrotic symptoms [1,5].

The long-term goal of research into bacterial canker of *Prunus* is to identify the genes essential for host colonisation and symptom development during the different phases of the bacterial infection cycle [5]. In the pathovars of *P. syringae*, it has been established that effector proteins secreted into plant cells by the type III secretion system (T3SS) and also low-molecular-weight toxins have key roles in the suppression of the plant’s immune responses, allowing successful colonisation of the apoplast to take place [6,7,8]. The identification of the T3SS was achieved by transposon mutagenesis of *P.s.* pv *phaseolicola* and complementation analysis of mutants recovered that had lost the ability to induce a hypersensitive reaction as well as pathogenesis [9].

The mutant screening approach has been the cornerstone of genetic dissection of pathogenicity and has been used with numerous plant pathogenic bacteria [10,11]. In addition to effectors and toxins, many other genes with less clearly defined roles in virulence have been found to influence disease outcomes. For example, in a study of *P. cannabina* pv. *alisalensis* (*P. cal*), Sakata et al. [12] tested 1040 Tn*5* mutants by dip inoculation onto cabbage plants. *P. cal* causes bacterial blight of *Brassicaceae,* an economically important crop which includes plants such as cabbage, broccoli, cauliflower and mustards. A total of 53 mutants showed decreased virulence and further tests revealed that 31 of these mutants also exhibited decreased virulence on oat seedlings. The potential virulence factors identified included not only genes encoding the T3SS, but also membrane transporters and enzymes involved in amino acid metabolism. Manoharan et al. [13] selected 106 out of 1920 Tn mutants from *P.s.* pv. *phaseolicola* using an in vitro screen looking for changes in motility, colony formation, and adhesion, which were proxies for infection, microcolony formation and cell adhesion. A number of mutants with altered motility bearing mutations in genes encoding various parts of the flagellum were found to lose pathogenicity. However, a mutation in a gene encoding an unidentified conserved hypothetical protein caused a positive increase in bacterial multiplication in planta.

A new approach to mutagenesis, allowing rapid identification of genes improving pathogenic fitness, is transposon sequencing (Tn-seq). This method is based on recent developments in high-throughput sequencing platforms and involves creation of a saturated Mariner transposon insertion library followed by deep-sequencing of populations of bacteria to identify the genes maintained during infection [14]. The application of Tn-seq has allowed genome-wide identification of genes in *P.s*. pv. *syringae* B728a required for fitness during colonisation of the bean leaf surface and inside the plant [15]. Genes within the functional categories of amino acid and polysaccharide biosynthesis contributed most to fitness, both on the leaf surface (epiphytic) and in the leaf interior (apoplast), while genes involved in the T3SS and also syringomycin toxin synthesis were important in the apoplast. Of the secreted type III effectors, disruption of *hopAB1* had the greatest effect on intercellular colonisation. Numerous other genes that had not been previously associated with in planta growth were also required for maximum epiphytic or apoplastic fitness. In short, mutagenesis experiments have shown that there is more to the life of a plant pathogen than the T3SS, effectors, and toxins.

The inoculation of immature cherry fruit provides a rapid test of *P.s.* pathogenicity that is well suited for mutant screening experiments [1]. Here, we describe experiments using the fruit assay as a primary screen of Tn mutants of each of the major cherry canker pathogens *Pss*, *Psm* R1 and *Psm* R2. Mutants with reduced and also enhanced virulence recovered from the fruit assay were further characterised by in vitro growth parameters including biofilm formation [13] and leaf and cut shoot pathogenicity tests. The mutated genes in a selection of mutants with altered pathogenicity were identified by sequencing and complementation experiments. Intriguingly, mutation of the effector *hopAU1* in *Psm* R2 was found to lead to increased symptom development in all assays.

## 2. Materials and Methods

### 2.1. Bacterial Strains and Growth Conditions

Bacterial strains and plasmids used in this study are listed in Table 1. *Escherichia coli* strains were grown at 37 °C in Lysogeny Broth (LB, Difco, Detroit, MI, USA) medium supplemented with 15 g/L Bacteriological No.1 agar (Oxoid, Altrincham, Cheshire, UK) and *Pseudomonas* strains were grown at 25 °C on Kings medium B (KB, Difco, Detroit, MI, USA) or in LB broth. Selected mutants were grown on M9 minimal media supplemented with 20% glucose to test for auxotrophy. Antibiotics were used at the following concentrations (μg/mL): gentamycin (Gm) 10, kanamycin (Km) 50, rifampicin (Rif) 10, and nitrofurantoin (NF) 100.

### 2.2. Transposon Insertion Library Construction

Libraries of transposon (Tn) mutants were generated using *Psm* strains MH001 and 5244 and *Pss* strain 9644 (recipient strains) following bi-parental mating with *E. coli* S17-1 λpir carrying IS-Ω-Km/hah (donor strain) [17]. Essentially, 500 μL of an overnight culture was inoculated into 10 mL of LB broth and incubated for 2 h (donor strain) or 4 h (recipient strain). Following incubation, 30 μL of donor was mixed with 100 μL of an overnight culture of the required recipient strain and the mixture transferred to the centre of a LB plate before incubation at 30 °C for 48 h. This conjugation mixture was then diluted, plated on KB + Km + NF or Rif and incubated at 25 °C for 72 h. Single-transformation mutants were selected and inoculated on KB + Km in a 48 colony grid pattern and numbered. The locations of transposons in the genome were identified using Arbitrary Primed (AP)-PCR and DNA sequencing (Eurofins Genomics) [13] (see Table S1 for primer sequences). The resulting sequences were analysed using the BLAST programme from the NCBI.

### 2.3. Screening of Transposon Insertion Libraries

#### 2.3.1. Pathogenicity Testing

The initial screen was performed on detached unripe cherry (*Prunus avium*) fruit, as described in Hulin et al. [1]. Green cherry fruit, cultivar Sweetheart, were inoculated with individual colonies from 48 colony replica plates using a sterile cocktail stick. The fruit were placed on boards with ~200 pre-drilled holes and incubated at 23 °C, 80% humidity, 16/8 h light/dark cycle in a growth cabinet. The fruits were scored by eye for symptoms and photographed after 5 days. Scoring was as follows: wild type (WT, +), no lesions other than the inoculation wound (--), lesion formation less than in the wild type (-), increased symptoms (++), increased symptoms with signs of spread (+++). Detached 1–2-week-old leaves of cherry cultivar Sweetheart obtained from NIAB EMR were syringe infiltrated with 2 × 10^8^ CFU/mL bacterial suspension from an overnight broth culture. Six replicate leaves were inoculated per strain and leaves were placed in plastic trays on water agar layered with damp tissue paper and incubated at 23 °C 16/8 h light/dark cycle. Visual symptoms were recorded and photographed after 10 days.

For in planta growth analyses, dormant cherry shoots of susceptible cultivar Van were obtained from NIAB EMR. Shoots were sterilised in 5% hypochlorite for 5 min before being washed 3 times in tap water and then air dried overnight. Shoots were cut to 10 cm in length and a sterile scalpel used to carve an ‘I’ shape into the bark, which was then peeled back and the shoot inoculated with 8 × 10^7^ cfu/mL bacterial suspension. This was allowed to soak in for 5 min before the bark was folded back down and the wound wrapped in parafilm. The shoots were stood in 2 cm water and incubated at room temperature for 5, 10 and 14 days. A sterile scalpel was then used to harvest the inoculated area (10 mm long by 10 mm wide and 3 mm down into the tissue), and this was homogenised in ¼ Ringers solution before being diluted and spread plated onto KB plus appropriate antibiotic selection. Colonies were counted after three days.

#### 2.3.2. Colony Morphology

Tn libraries were replica plated onto KB agar with the WT control strain in position one. Each library plate was tested in triplicate and plates were incubated at 25 °C for three days before visual observation of the colony phenotypes.

#### 2.3.3. Biofilm Attachment

The biofilm attachment assay was modified from Spiers et al. [19]. Tn library plates were replica plated into a 96-well microtiter plates containing 200 μL LB broth + Km. The WT control was tested without Km in plate position one. Plates were incubated at 25 °C for seven days without shaking. After seven days, the bacteria attached to the microtiter plates were stained with 1% crystal violet (CV). The density of the eluted CV was determined at OD_570_ using a microtiter plate reader (FLUOstar OPTIMA, BMG Labtech, Ortenberg, Germany). To test individual mutants, strains were incubated in 10 mL of LB broth + Km static at 25 °C for seven days. After seven days, attached bacteria were stained as described above with 1 mL of 1% CV.

#### 2.3.4. In Vitro Growth Rate

Initial growth assays were carried out in 96-well plates containing 200 µL LB broth, inoculated directly from plate culture with a replica plater and incubated for 8 h shaking. The cell density was determined at OD_600_ using a microtiter plate reader (FLUOstar OPTIMA, BMG Labtech, Ortenberg, Germany). Any mutants that showed a statistically significant growth rate compared to their respective WT strain as determined by Student’s *t*-test (*p* < 0.05) were repeated in triplicate. Individually selected mutants were grown overnight in LB broth + Km and diluted to 8 × 10^8^ CFU/mL (OD_600_ 1.0). Cells (100 μL) were subcultured into 10 mL fresh LB broth and the optical density measured and recorded. All cultures were incubated at 25 °C with shaking (10 g) and their optical densities measured and recorded after 8 h.

#### 2.3.5. Swarming Ability

Selected mutants were tested for swarming motility individually. Mutants and WT strains were spotted into the centre of a 0.3% LB agar plate. Each mutant was tested in triplicate and plates were incubated at 25 °C for 72 h before visual observation and measurement of the colonies’ swarming motility.

### 2.4. Cloning and Complementation

Ectopic complementation of selected Tn mutants was carried out following PCR amplification of the disrupted genes including both start and stop codons and a 100 bp region upstream of the start codon to include likely promotor sequences (see Table S1 for primer sequences) and insertion of the resulting fragment into TOPO pCR2.1 (Invitrogen) by TA cloning following the manufacturer’s instructions. The resulting construct was partially digested with *Eco*R1 and the fragment of the correct size was ligated into broad host range vector pBBR1MCS-5, allowing expression from the Tn*3* or Tn*7* promoters [18]. These constructs were introduced into their respective mutant strain via electroporation, as described by Keen et al. [20]. An empty vector was also transformed into the mutants as a control.

## 3. Results

### 3.1. Pathogenicity Screening of Pseudomonas syringae Transposon Mutants

**Figure 1:**
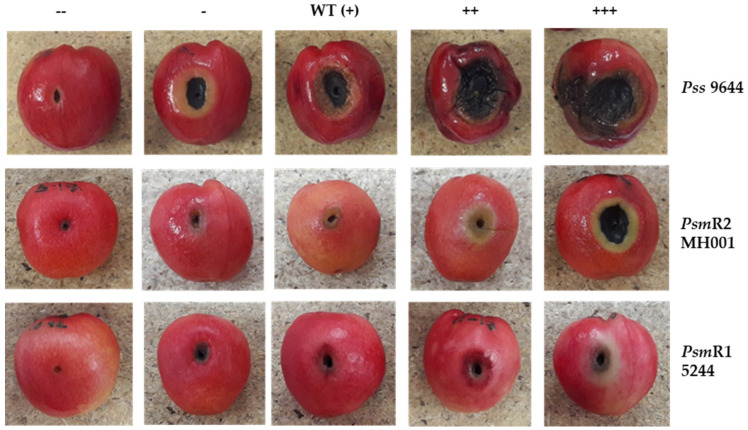
Screening of *Pseudomonas* transposon disruption mutants for changes in pathogenicity on cherry fruit. Transposon mutants and associated wild-type (WT) strains were inoculated into immature cherry fruit and photographed after 5 days. Symptoms were scored as WT (+), as displayed by the wild-type strain; --, no symptoms; -, reduced symptoms; ++, increased symptoms; +++, increased symptoms with clear signs of spread. *Psm*, *P. syringae* pv. *morsprunorum*; *Pss*, P. *syringae* pv. *syringae*.

Libraries of transposon mutants were made of the three pathogenic strains—*Pss* 9644, *Psm* 5244 and *Psm* MH001. Due to the relative inefficiency of the conjugation process, and a desire to test the proof of principle of this approach, 940 Tn mutants per library were chosen. The libraries were screened for changes in pathogenicity on unripe detached cherry fruit. Each insertion mutant was picked directly from a colony using a sterile cocktail stick and stabbed into fruit. Each mutant was tested three times and the symptoms scored and photographed after five days incubation at 23 °C. Symptoms were scored compared to WT, Figure 1 illustrates the range of symptoms observed.

A total of 76, 53 and 113 mutants of *Pss* 9644, *Psm* 5244 and *Psm* MH001, respectively, showed changes in pathogenicity compared to WT. Numbers of individual Tn mutants that show changes in pathogenicity are given in Table 2.

### 3.2. In Vitro Screening of Transposon Mutants

**Figure 2:**
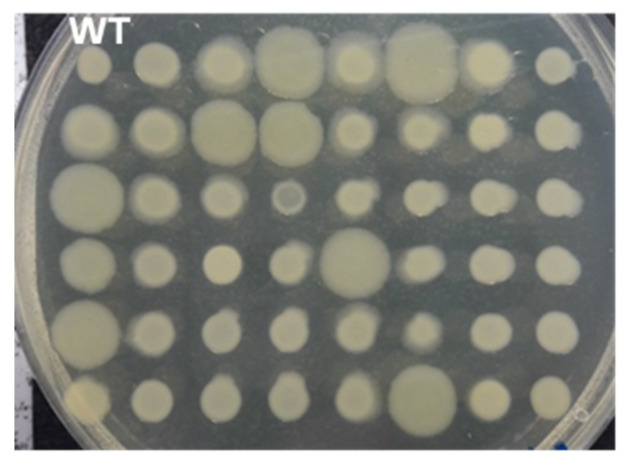
Screening of *Pseudomonas syringae* transposon disruption mutants for changes in colony morphology. Tn mutant colonies (48) were inoculated onto standard agar plates and changes in colony morphology recorded after 72 h, Colonies were replica plated in a 48 grid pattern with the wild-type (WT) strain at position 1. Plate contains 47 *P. syringae* pv. *morsprunorum* R2 MH001 transposon mutants.

**Figure 3:**
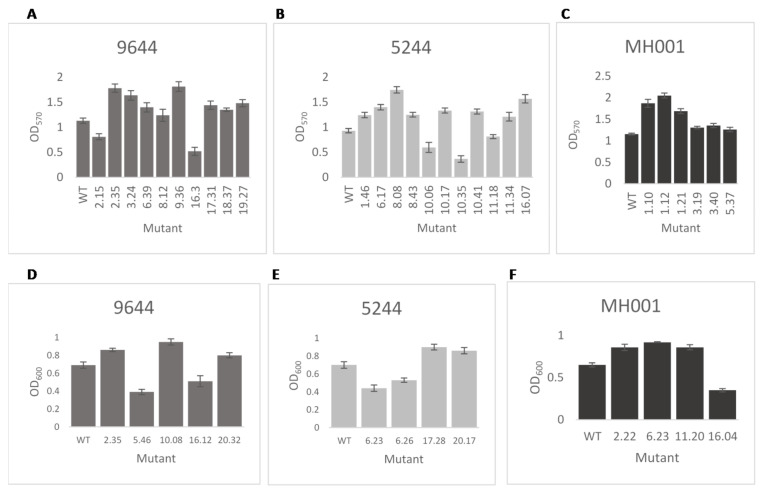
Selected *Pseudomonas syringae* transposon disruption mutants in biofilm attachment assay and in vitro growth rate. Transposon mutants and wild-type strain (WT) were cultured static in 10 mL broths. After seven days, crystal violet was used to measure (OD_570_) the attachment of the cells to the culture vessel surface (**A**–**C**). Differences in in vitro growth were analysed after 8 h growth in LB broth (**D**–**F**). (**A**,**D**) *P. syringae* pv. *syringae* 9644; (**B**,**E**) *P. syringae* pv. *morsprunorum* R1 5244; (**C**,**F**) *P. syringae* pv. *morsprunorum* R2 MH001. The mutants displayed show a statistically significant difference to their respective WT strain determined, *p* < 0.05 as assessed by Student’s *t*-test. Error bars represent the standard error of the mean of three biological replicates.

Transposon insertions may have had an impact on bacterial metabolism, affecting growth or the ability to colonise and/or attach to the plant cell within the apoplast, thus the libraries of mutants from each pathogen were tested for in vitro growth characteristics—colony size, biofilm formation and growth rate in liquid culture. A number of alterations from the wild type were recorded for each strain. Results from a selection of mutants, illustrating the variations observed in mutants of each pathogen, are shown in Figure 2 and Figure 3. Mutations of *Psm* MH001 only produced mutants with an increased adherence ability.

The numbers of mutants with each phenotype from in vitro assessment and pathogenicity scores were compared determine whether changes in pathogenicity related to changes in phenotype (Table 2). Mutants with higher or lower growth rates in vitro were similarly more or less pathogenic in the cherry fruit assay. A similar trend was noted for biofilm formation, although there were exceptions, for example, of the three *Psm* 5244 mutants with reduced biofilm formation, two recorded increased symptom scores. Results recorded for all the assays for each mutant can be found in Table S2.

### 3.3. Selection of Transposon Mutants for Further Analysis

From the results of the phenotypic screens, 18 mutants were selected for further analysis (Table 3). The focus was on mutants with altered virulence and included twelve (two *Pss* 9644 and 10 *Psm* MH001) that produced no disease symptoms on cherry fruit, but displayed WT colony morphology. Genomic analysis of the selected genes between strains showed that some of the genes are present in all three strains but some are only present in one or two strains (Table S3). Six mutants that displayed hypervirulence on cherry fruit were also selected for further analysis (four *Pss* 9644, one *Psm* 5244 and one *Psm* MH001).

### 3.4. Swarming Ability of Selected Transposon Mutants

The chosen 18 transposon mutants were subjected to a further phenotypic test to determine whether the insertions had affected their motility and hence their ability to disperse throughout the apoplast, which is expected to be an important factor in successful colonisation of the plant. Mutant and WT strains were spotted individually onto 0.3% soft LB agar plates and incubated for 72 h at 25 °C, before being visually compared and measured (Table 3). Swarming ability was altered in five out of the 18.

### 3.5. Identification of Disrupted Genes in Selected Transposon Mutants

The gene disrupted in each mutant was identified by AP-PCR and sequencing using primers located in the transposon and semi-random primers for the chromosome (Table 3). As a number of mutations were identified in genes involved in metabolism all 18 mutants were tested for growth on M9 minimal medium. Absence of growth on M9 minimal medium was observed for 9644 mutant strain 3.15 (Tn located in a serine hydroxymethyltransferase 2) and MH001 mutant strain 4.02 (Tn located in an ornithine carbamoyltransferase).

Taken together, even within the small sample of mutants analysed in depth, the mutations affecting virulence were found to be in genes involved in diverse functions including membrane transport, amino acid metabolism, DNA repair and primary metabolism. In addition, we found examples of mutations in genes involved in the T3SS (hrcS) and flagellar assembly (fliK) that led to loss of pathogenicity. By contrast, an insertion in the effector encoding gene hopAU1 led to increased virulence.

### 3.6. Complementation of Transposon Mutants

**Figure 4:**
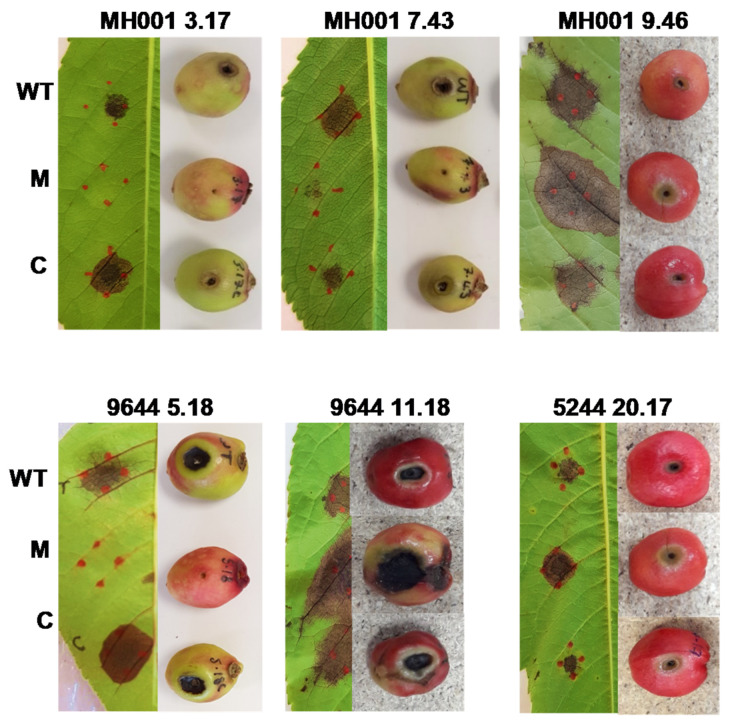
Complementation of selected *Pseudomonas syringae* transposon disruption mutants. Mutants that showed no disease symptoms (-) on cherry fruit but displayed wild-type (WT) characteristics in all other phenotypic assays, along with several mutants that displayed hypervirulence (+++) on cherry fruit were selected for complementation. The disrupted genes (3.17, *dnaJ*; 7.43, *hrcS*; 5.18, glycosyl transferase 1; 9.46, *hopAU1*; 11.18, *nhaA*; 20.17, *argE*) were cloned into a broad host range vector and transformed into their respective mutant strain. The WT, Tn mutant (M) and complemented strains © were tested on cherry fruit and cherry leaves to confirm restoration pathogeniciy in the complemented strains. Images displayed show 6 of the 18 complemented strains. However, in all cases, pathogenicity was restored on both fruit and leaves. Images of 9644—11.18 and 5244—20.17 are composites of three photographs.

To determine whether the wild-type phenotype could be restored, complementation experiments were carried out. Primers designed to include putative promoter regions, start and stop codons were used to amplify the genes from WT DNA of each strain. Functional copies of the disrupted gene were cloned into a broad host range vector pBBR1MCS-5 and electroporated into the matching Tn mutant. An empty vector was also transformed into all the mutants tested and used as a control. The WT, mutants, complemented strains and controls were then inoculated onto both detached cherry fruit and detached cherry leaves of cv. Sweetheart and incubated for five and ten days, respectively, at 23 °C. Figure 4 shows six examples of the observed phenotypes. In all 18 cases, the WT disease phenotype was restored by complementation in fruit and leaves. The empty vector control did not affect the mutant phenotype in any strain (data not shown).

**Figure 5:**
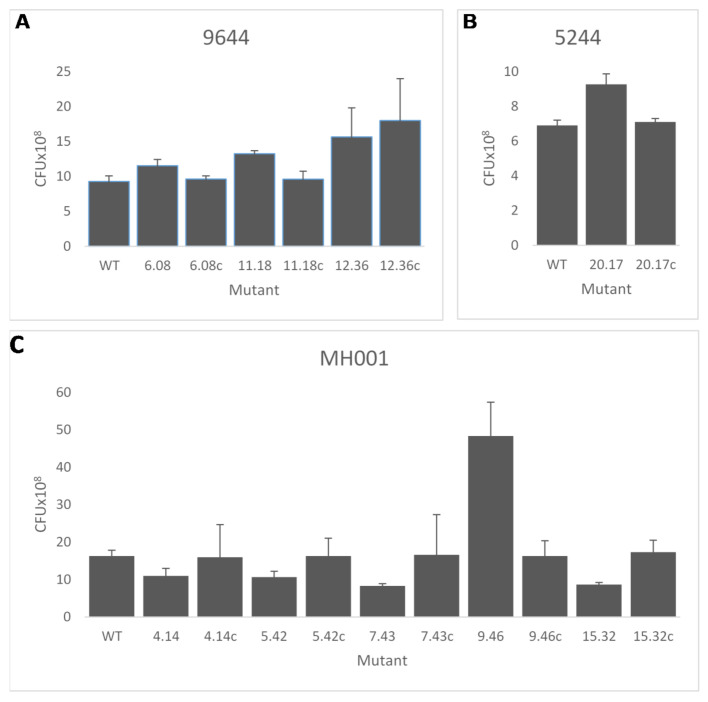
Growth of selected *Pseudomonas syringae* transposon disruption mutants and complemented strains in woody tissue. (**A**) *Pss* 9644 (6.08, pyridoxine/pyridoxamine 5′-phosphate oxidase; 11.18, *nhaA*; 12.36, arginine-ornithine antiporter). (**B**) *Psm* R1 5244 (20.17, *argE*). (**C**) *Psm* R2 MH001 (4.14, phosphoglycerate mutase, 2,3-bisphosphoglycerate; 5.42, DNA ligase D; 7.43, *hrcS*; 9.46, *hopAU1*; 15.32, *fliK*). Growth in cherry cv. Van shoots was assessed at 14 days. c, complemented strain. Error bars represent the standard error of the mean of three WT biological replicates. Figure shows only mutants that had significantly different growth compared to the WT strain determined as *p* < 0.05 by Student’s *t*-test.

Bacterial multiplication at inoculation sites was examined in all 18 mutants and their complemented strains using woody shoots of cherry cv. Van (this was the only susceptible cultivar available for use at the time). The complementation apparent in the fruit and leaf assays was reflected by restoration of bacterial populations to those recorded for the wild-type strains (Figure 5) for all except 9644—12.36 (arginine-ornithine antiporter) where the complementation failed to restore WT population levels in woody tissue.

## 4. Discussion

The development of disease-resistant cherry tree varieties is a central approach to reducing yield losses and securing fruit supply for the future. Key to developing such varieties is an understanding of how pathogens cause disease and trigger resistance, especially when there is diversity in pathogen genotypes. To examine this, we used random transposon (Tn) mutagenesis in combination with a series of screens to identify mutants with altered phenotypes potentially associated with virulence in cherry canker pathogens *Pss* and *Psm*. Overall, we screened 940 colonies from each of three Tn libraries of *Pss* 9644, *Psm* R1 5244 and *Psm R2* MH001. Combining the results of the various tests, we chose 18 mutants to focus on, including six that appeared to be hypervirulent on cherry fruit. When comparing the 18 genes to two other small-scale Tn screens in *P. syringae* [13] and *P. cannabina* [12] as well as a more in-depth Tn screen [15], we were able to see overlaps in the general classes of genes observed to be important (e.g., amino acid metabolism and transport, T3SS, cofactor metabolism) as well as some specific matches (e.g., *glyA*, *dnaJ*, acetolactate synthase). These demonstrate some commonalities in genes that play important roles in virulence as well as the expected diversity observed due to the influence of host and environment on pathogen evolution.

Of the six mutants that showed an increase in disease symptoms on cherry fruit, five also showed increased multiplication in planta. These include *Pss* 11.18 and 12.36, which had WT colony morphology and WT in vitro growth, and the mutated genes encoding for Na(+)/H(+) antiporter NhaA and an arginine-ornithine antiporter ArgE, respectively. An antiporter is a membrane protein that actively transports two or more different molecules through the plasma membrane in opposite directions. Na+/H+ antiporters catalyse the efflux of Na+ in exchange for H+ in order to regulate intracellular pH. Most bacteria contain between five and nine different Na+(K+)/H+ antiporters [21], which may explain how mutation of *nhaA* did not lead to a reduction in growth in vitro. Herz et al. [22] created a triple mutant of three Na+/H+ mutants in *Vibrio cholerae* and found no significant reduction in exponential growth in high pH environment. One theory to explain this phenomenon is that the bacterium may employ alternative regulatory strategies, for example functional redundancy or compensatory upregulation mechanisms, to rescue negative effects caused by the single-antiporter gene disruptions [23]. Three mutants involved in arginine metabolism were observed—two showing hypervirulence and one loss of virulence. Notably, arginine metabolism genes (*argA* and *argB*) were identified in a transposon mutagenesis screen of the olive knot pathogen, *P. savastanoi* pv. *savastanoi* [24]. As with olive, we know nothing about the cherry metabolome and the status of arginine levels, unlike tomato apoplast where levels are very low [25]. Together, these observations indicate a deeper metabolome analysis of cherry is warranted that examines how the pathogen interacts with arginine and how it influences disease. By contrast, *Pss* 6.08 (Pyridoxine/pyridoxamine 5′-phosphate oxidase) and *Psm* 5244 20.17 (Acetylornithine decacetylase) showed an increase in disease symptoms and an increase in in planta growth but also had a larger than WT colony morphology and *Psm* 5244 20.17 also had higher in vitro growth than WT but, by contrast, a reduced swarming ability (65 ± 6). Pyridoxine/pyridoxamine 5′-phosphate oxidase is part of the vitamin B6 biosynthesis pathway and acetylornithine decacetylase is an enzyme in step 1 of the sub-pathway that synthesises l-ornithine from *N*(2)-acetyl-l-ornithine (linear).

An increase in disease symptoms and multiplication in planta was also recorded for *Psm* MH001 9.46 but this mutant had WT in vitro characteristics. The transposon located *Psm* MH001 9.46 disrupts the type III effector *hopAU1* which is present in most strains of *Psm*R1 and R2, but not the *Pss* strains pathogenic to cherry [1]. The function of HopAU1 has not been determined but the protein has been shown to be localised to the plant cytoplasm and excluded from the nucleus in *Psa* [26]. It is also necessary for growth at later stages of infection by *Pph* strain 1448A in bean [27]. Why a disruption in the genes responsible for a type III effector, metabolic enzymes and the antiporter genes discussed above would lead to increased in planta growth and enhanced disease symptoms is unclear but highlights the complexity of the control of virulence in these pathogens. It may be that deletion of these genes leads to advantage as a plant pathogen under laboratory conditions but would be detrimental to the bacterium in the field. There is likely to be a delicate balancing act between being a more aggressive pathogen and surviving under other circumstances. It may also be possible that the presence of a functional HopAU1 protein and the production and release of other metabolites from the cell activates some level of host resistance that reduces pathogen fitness in planta and this may help to explain why *hopAU1*, for example is absent from certain cherry pathogens like *Pss*. This suggests a further analysis using experimental evolution approaches may help to understand the nuances underpinning selective pressure to maintain this gene in *Psm* in the face of reduced fitness, especially if it reveals a weak but quantifiable host resistance.

In contrast to the above mutants the final hypervirulent mutant *Pss* 9644—1.38 showed increased symptoms on cherry fruit but no increase in multiplication in woody tissue. The mutation in 1.38 is within an adenine DNA glycosylase, a member of a family of enzymes involved in base excision repair. The increased symptom development caused by the mutant may be due to increased toxin or degradative enzyme production or activity, but the outcome clearly does not improve overall fitness of the pathogen as measured by growth.

Of the 12 mutants that produced no disease symptoms on cherry fruit but displayed WT colony morphology and WT in vitro growth, only four (all *Psm* MH001) showed decreased growth in cherry stems. One of these *Psm* MH001 7.43 encodes the T3SS protein HrcS. HrcS codes for an inner membrane-spanning protein of the T3SS and is crucial for pathogenicity [28], so we would expect disruption of it to cause a decrease in disease. Another mutant causing no disease is *Psm* MH001 5.42, which encodes for a DNA ligase D (LigD), an ATP-dependent enzyme that joins breaks in the phosphodiester backbone of DNA that occur during replication and recombination and as such they are essential for DNA replication and repair in all organisms. *Psm* MH001 4.14 is a phosphoglycerate mutase (PGM), an enzyme that catalyses step 8 of glycolysis. PGM catalyses the internal transfer of a phosphate group from C-3 to C-2, which results in the conversion of 3-phosphoglycerate (3PG) to 2-phosphoglycerate (2PG) through a 2,3-bisphosphoglycerate intermediate. Morris et al. [29] demonstrated that functional PGM is essential for the growth and pathogenicity of *P.s*. pv. *tomato* on its host as a mutation in PGM renders the strain unable to grow and elicit disease symptoms on tomato seedlings. *Psm* MH001 15.32 is a disruption to the flagellar hook-length protein FliK [30]. Flagella confer motility to a number of bacteria [31] and discovery that flagellar genes are involved in virulence to the plant is not unexpected as it has been recorded in a number of plant pathogens [32,33]. FliK has previously been identified in another *P. syringae* Tn screen as a mutant that had a reduced ability for swarming in the *Pph* strain 1448A bean screen [13]. Four of the mutants that produced no disease also showed a reduced swarming ability—these were 9644—3.15, a serine hydroxymethyltransferase 2, and the three MH001 mutants discussed above (4.14, 5.42 and 15.32). 15.32 was particularly reduced in swarming (32 ± 3%) as could be expected of a flagellar mutant. Three of the 12 mutants—9644 3.15, a serine hydroxymethyltransferase 2; 9644—5.18, glycosyl transferase family 1; and MH001—3.17, molecular chaperone DnaJ—were also identified by Helmann et al. [15] as genes that when disrupted reduced the fitness of *Psy* B728a in apoplastic fluid by <−2.

In all 18 cases, the WT disease phenotype was restored in fruit and leaves by complementation and 17 of the complemented mutants restored their growth in woody tissue also. However, we failed to restore the WT woody tissue growth phenotype in mutant 9644 12.36, indicating that the insertion may be having a polar effect on a surrounding gene. The IS-Ω-Km/hah transposon carries a strong *nptII* promotor on its inside end (IE) [17]. The location of the insertion in mutant 12.32 indicates that the *nptII* promotor may be activating the adjacent gene BKM12_0040 an arginine deiminase, which may explain why complementation was not possible in the stem inoculations.

Although this study used a small sample size, it has identified a number of genes of potential interest with widely different functions. An unexpected observation was that the mutation of six genes caused the parent strain to become hypervirulent on cherry fruit and leaves, four of which became hypervirulent in woody tissue as well. Hypervirulence has been observed following single-gene deletions in a range of pathogenic microorganisms. Brown et al. [34] identified 112 hypervirulent mutations from 37 pathogen species using the pathogen-host interaction database. The 112 genes identified were from across kingdoms, in many cases conserved and came from a number of molecular, biochemical and cellular pathways. If hypervirulence mutations can be selected for in a population without any subsequent cost to the bacteria, it is possible that more virulent forms of these pathogens could evolve. Thus, understanding the potential barriers and drivers of this process becomes important to ensure that management approaches do not inadvertently promote the emergence of more pathogenic strains.

## Figures and Tables

**Table 1:** Bacterial strains and plasmids.

Strain	Description	Reference
*Pseudomonas*		
*Psm* 5244	NF^R^. Race 1	[16]
*Psm* MH001	NF^R^. Race 2	[16]
*Pss* 9644	Rif^R^. Race 1	[16]
*E. coli*		
pCR2.1 TOP10F	Competent cells	Invitrogen, UK
S17-1 λpir	KM^R^. Containing plasmid pSCR001	[17]
Plasmids		
pBBR1MCS-5	Gm^R^. Broad host range cloning vector	[18]
pSCR001	Km^R^. Carries IS-Ω-Km/hah transposon	[17]
pCR2.1	Km^R^, Amp^R^. Cloning vector	Invitrogen, UK

**Table 2:** Numbers of transposon disruption mutants selected with modified phenotypes after screening 940 Tn insertions in each strain.

**Strain**	**Pathogenicity Phenotype Compared to WT (+)**					
	**--**	**-**	**++**	**+++**		
**9644**	7	30	25	14		
**MH001**	11	22	53	27		
**5244**	25	8	17	3		
**Morphology**						
	**Larger**	**Pathogenicity**	**Smaller**	**Pathogenicity**	**Irregular Shape**	**Pathogenicity**
**9644**	24	1 reduced 4 increased	34	9 reduced 2 increased	14	4 reduced 1 increased
**MH001**	14	9 increased	15	1 reduced	0	-
**5244**	32	6 increased	37	20 reduced	5	3 reduced
**Biofilm**						
	**Increased**	**Pathogenicity**	**Decreased**	**Pathogenicity**		
**9644**	9	1 reduced 4 increased	2	2 reduced		
**MH001**	6	2 reduced 4 increased	0	-		
**5244**	8	3 reduced 5 increased	3	2 increased		
**In Vitro Growth**						
	**Increased**	**Pathogenicity**	**Decreased**	**Pathogenicity**		
**9644**	3	2 increased	2	2 reduced		
**MH001**	3	3 increased	1	1 reduced		
**5244**	2	2 increased	2	2 reduced		

9644, *P. syringae* pv. *syringae*; 5244 and MH001, *P. syringae* pv. *morsprunorum*. WT, wild type. Pathogenicity, response on immature cherry fruit; --, no symptoms; -, reduced symptoms; ++, increased symptoms; +++, increased symptoms with clear signs of spread.

**Table 3:** Characteristics of selected *Pseudomonas syringae* transposon mutants.

Mutant Number	Symptoms ^1^	Swarming % WT	Gene Name/ Description	Biological Process	Locus Tag
***Pss* 9644**					
1.38	+++	WT	Adenine DNA glycosylase	Base excision repair	BKM12_20200
3.15	-	92 ± 3	Serine hydroxymethyltransferase 2	Amino acid biosynthesis	BKM12_25860
5.18	-	WT	Glycosyl transferase family 1	*N*-glycan biosynthesis	BKM12_16730
6.08	+++	WT	Pyridoxine/pyridoxamine 5′-phosphate oxidase	Vitamin B6 biosynthesis	BKM12_05440
11.18	+++	WT	Na(+)/H(+) antiporter, *nhaA*	Sodium ion transport	BKM12_00120
12.36	+++	WT	Arginine-ornithine antiporter, *argE*	Arginine deiminase pathway	BKM12_00245
***Psm* R1 5244**					
20.17	+++	65 ± 6	Acetylornithine deacetylase	Arginine biosynthesis	BKM19_RS02785
***Psm* R2** **MH001**					
3.17	-	WT	Molecular chaperone, *dnaJ*	DNA replication	BKM03_RS28190
3.27	-	WT	MFS transporter	Transmembrane transporter activity	BKM03_RS09500
4.02	-	WT	Ornithine carbamoyltransferase	Arginine biosynthesis	BKM03_RS22585
4.14	-	87 ± 2	Phosphoglycerate mutase, 2,3-bisphosphoglycerate	Glycolysis pathway	BKM03_RS28005
5.42	-	78 ± 5	DNA ligase D	DNA replication	BKM03_RS11910
6.37	-	WT	Beta-glucosidase	Cellulose catabolic process	BKM03_RS12410
7.43	-	WT	T3SS protein, *hrcS*	Bacterial secretion system	BKM03_RS07835
8.11	-	WT	Cu+ exporting protein	Metal ion binding	BKM03_RS10500
9.16	-	WT	Acetolactate synthase	Amino acid biosynthesis	BKM03_RS05770
9.46	++	WT	Type III effector, *hopAU1*	Virulence	BKM03_RS30415
15.32	-	32 ± 3	Flagellar hook-length protein, *fliK*	Flagellar assembly	BKM03_RS07850

^1^ Pathogenicity on cherry fruit; --, no symptoms; ++, increased symptoms; +++, increased symptoms with clear signs of spread. Biofilm-forming ability and in vitro growth all as WT except mutant 5244—20.17 displayed in vitro growth of 121 ± 8% and mutants 9644—6.08 and 5244—20.17 had larger colony morphology.

## Data Availability

Data underlying this article can be accessed at http://researchdata.uwe.ac.uk/627/.

## Supplementary Materials

The following are available online at https://www.mdpi.com/article/10.3390/microorganisms9061328/s1, Table S1: PCR primers used in this study; Table S2: Screening results for all Tn mutants; Table S3: Presence of selected mutant genes in the other strains used in this study.
