# Supplementary material for: Transposon Mutagenesis of Pseudomonas syringae Pathovars syringae and morsprunorum to Identify Genes Involved in Bacterial Canker Disease of Cherry
Source: Microorganisms. 2021 Jun 18;9(6):1328. doi: 10.3390/microorganisms9061328 (PMC8234094; doi:10.3390/microorganisms9061328)
Supplement: Supplementary file 1 [file microorganisms-09-01328-s001.zip › Table S1 PCR primers used in this study.pdf]

Table S1. PCR Primers used in this study.

Primer name	Target gene or location	Sequence 5'-3'
9644-1.38	Adenine DNA glycosylase	CGACTCACATCTCGACGAG<br>TTGCGGCACATTACGGTGCG
9644-3.15	Serine hydroxymethyltransferase 2	TTGAGGACTGCCATGTTCAG<br>CTCATCAAGCGCCGTAAACC
9644-5.18	Glycosyl transferase family 1	TTTCATCAGCAGGACGCTCA<br>CGTTGCTGTTGTGGGTTCAG
9644-6.08	Pyridoxine/pyridoxamine 5'-phosphate oxidase	TCGATCCGTGCCCTCGCAG<br>GCTCAGACGATTGGCCAAAC
9644-11.18	Na(+)/H(+) antiporter NhaA	GCAGCGATCAATCGCTCCAG<br>ATAATCGAACCGTGGAGAACC
9644-12.36	Arginine-ornithine antiporter	CGATAAGGGTCAGCAGGCC<br>TCGCTCATGTCGTCGACCTG
5244-20.17	Acetylornithine decacetylase	TTACAGGAAGCGCCCATGCC<br>GTTGATAGTACCGGGCTCAC
MH001-3.17	Molecular chaperone DnaJ	CCACAAACAGAAAATGCCCTC<br>ATGGACAGCTAGTGCAATGC
MH001-3.27	MFS transporter	GGACGATGAGCCAACAGTC<br>TATGGAGCTGAGTATGCAGG
MH001-4.02	Ornithine carbamoyltransferase	AGGTAGTTAATCATGAACGCC<br>TCAGCAGCAACTGACTCATG
MH001-4.14	Phosphoglycerate mutase, 2,3-bisphosphoglycate	CATCGAGTGGGTTCTATGAC<br>GCTATAGGCCGGTTTTCACC
MH001-5.42	DNA ligase D	TACGGAGAAACCGGCCATGG<br>CCGCCTGATTTCAAGGCCG
MH001-6.37	Beta-glucosidase	TCATTCAGCGCGAGGCTTAC<br>GAACAAGCGAAAGATGATAGG
MH001-7.43	HrcS T3SS protein	AACGCCAACGCTTCCATGAC<br>CGGCTCAGCCATGATCATG
MH001-8.11	Cu+ exporting protein	AGAACTCATGACCGATGAAGC<br>ATGGCATGGCACAGGAACAATC
MH001-9.16	Acetolactate synthase	AATGGCCTGAACGGCCACG<br>AAACGCGACCGATTGGCCG
MH001-9.46	Type III effector HopAU1	GGGCAAGAAGCGTCATGCTC<br>CCTTATTCCTGCTGCGTGCG
MH001-15.32	Flagellar hook-length protein FliK	CAAGGCGCTCATGCTGAAAG<br>AGGTTAAGTGACGATGCCGG
TnphoAII-F	IS-Ω-Km/hah specific primer	GTGTGCAGTAATATCGCCCTGAGCA
CEKG2A	IS-Ω-Km/hah arbitrary primer	GGCCACGCGTCGACTAGTACNNNNNNNNNNNAGAG
CEKG2B	IS-Ω-Km/hah arbitrary primer	GGCCACGCGTCGACTAGTACNNNNNNNNNNNACGCC
CEKG2C	IS-Ω-Km/hah arbitrary primer	GGCCACGCGTCGACTAGTACNNNNNNNNNNNGATAT
Hah-1-F	IS-Ω-Km/hah specific primer	ATCCCCCTGGATGGAAAACGG
CEKG4	IS-Ω-Km/hah arbitrary primer	GGCCACGCGTCGACTAGTAC
