# Supplementary material for: Transposon Mutagenesis of Pseudomonas syringae Pathovars syringae and morsprunorum to Identify Genes Involved in Bacterial Canker Disease of Cherry
Source: Microorganisms. 2021 Jun 18;9(6):1328. doi: 10.3390/microorganisms9061328 (PMC8234094; doi:10.3390/microorganisms9061328)
Supplement: Supplementary file 1 [file microorganisms-09-01328-s001.zip › Table S3 Presence of selected mutant genes in other strains.pdf]

Table S3. Presence of selected mutant genes in the other strains used in this study.

**Mutant number**		
***Pss* 9644**	**Present in MH001**	**Present in 5244**
1.38	Y	Y
3.15	Y	Y
5.18	N	Y
6.08	Y	Y
11.18	Y	Y
12.36	N	N
***Psm* R1 5244**	**Present in MH001**	**Present in 9644**
20.17	Y	Y
***Psm* R2 MH001**	**Present in 5244**	**Present in 9644**
3.17	Y	Y
3.27	Y	Y
4.02	Y	Y
4.14	Y	Y
5.42	Y	Y
6.37	N	Y
7.43	Y	Y
8.11	Y	Y
9.16	Y	Y
9.46	N	N
15.32	Y	Y

Y: Yes, N: No. Analysis carried out using NCBI Blast. *Psm*; *Pseudomonas syringae* pv. *morsprunorum*, *Pss*; *P. syringae* pv. *syringae*.
